# Hybrid black silicon solar cells textured with the interplay of copper-induced galvanic displacement

**DOI:** 10.1038/s41598-017-17516-6

**Published:** 2017-12-07

**Authors:** Jheng-Yi Li, Chia-Hsiang Hung, Chia-Yun Chen

**Affiliations:** Department of Materials Science and Engineering, National Cheng Kung University, Tainan, 701 Taiwan

## Abstract

Metal-assisted chemical etching (MaCE) has been widely employed for the fabrication of regular silicon (Si) nanowire arrays. These features were originated from the directional etching of Si preferentially along <100> orientations through the catalytic assistance of metals, which could be gold, silver, platinum or palladium. In this study, the dramatic modulation of etching profiles toward pyramidal architectures was undertaken by utilizing copper as catalysts through a facile one-step etching process, which paved the exceptional way on the texturization of Si for advanced photovoltaic applications. Detailed examinations of morphological evolutions, etching kinetics and formation mechanism were performed, validating the distinct etching model on Si contributed from cycling reactions of copper deposition and dissolution under a quasi-stable balance. In addition, impacts of surface texturization on the photovoltaic performance of organic/inorganic hybrid solar cells were revealed through the spatial characterizations of voltage fluctuations upon light mapping analysis. It was found that the pyramidal textures made by copper-induced cycling reactions exhibited the sound antireflection characteristics, and further achieved the leading conversion efficiency of 10.7%, approximately 1.8 times and beyond 1.2 times greater than that of untexturized and nanowire-based solar cells, respectively.

## Introduction

Given the growing demand of renewable energy in last decade, silicon (Si)-based photovoltaic (PV) devices, a sustainable and clean approach that enabled to convert light into electricity have possessed the merits for electricity generation, and continued dominating the market share of renewable technique^1,2^. In practical aspect, extent of energy amortization of Si-based solar photovoltaics remained critical, which significantly depended on the efficiency of energy extraction gained from PV modules, and at the same time, the manufacture and installation cost was also correlated^3^. As such, the improved fabrication technique that essentially met these two criteria was highly desirable. A technique referred as surface texturization was a prerequisite in the fabrication procedures of commercialized Si-based PV devices^4,5^. It specified the approach that introduced the roughened textures on the surfaces of solar cells, emerging a substantial reduction of light reflectance through either diminishing the discontinuity gap of refractive index between PV devices and surrounding media, or allowing the collection of solar light illuminated with large angles. Based on this concept, recent strategy of technical evolution focused on the development of special topographic architecture termed “black silicon”, which featured an ideal design for photovoltaics due to its remarkable light management properties covering the entire solar spectrum^6^. Black silicon was a type of low-dimensional textures behaving as a tailored dielectric media with graded refractive index falling between the surfaces of PV devices and air, contributing to an extremely low reflectivity of light through harvesting broadband wavelengths of light^7^.

Metal-assisted chemical etching (MaCE) emerged a promising technique that has been extensively employed for the creation of highly light-absorptive black Si^8,9^. In general, MaCE reaction involved with a series of electrochemical interactions between Si and aqueous electrolytes. The involving reactions were predominantly taken place in the presence of metal catalysts, where the induced dissolution rate of Si was more than hundred times beyond the Si surfaces without a metal coverage^10^. This feature resulted in the directional etching of Si right beneath the metal, leaving the remnant of Si skeletons in nanowire shape. Through MaCE technique, nanoscale features were created with well structural controllability and high-speed formation rate, but the etching profiles preferentially appeared to be one dimensional. This was because the movement of metals, including gold (Au), silver (Ag), platinum (Pt) and palladium (Pd) utilized for initiating the catalytic etching tended to proceed directionally along <100> orientations^8,11,12^, whose involved back-bond strength was lowest in Si crystals. Such fundamental restriction limited the shape diversity of etching profiles in a controlled manner while utilizing a MaCE technique. More critically, the great challenge upon cell construction of PV devices remained in the creation of uniform *p*-*n* heterojunction onto one-dimensional nanotextures owing to the nature of high aspect ratio. This might further cause the fabrication complexity and thereby strongly hindered their practical applications on the realization of high-performance solar cells.

Taking together, our strategy was to develop a single-step etching technique that enabled to prepare the regular textures with pyramidal shapes rather than the conventional one-dimensional nanotextures; instead such introduced surface structures still maintained the sound light-trapping capability. Different from the developed MaCE method where the etching reaction was the major motive contributing to the generation of eventual structures, our cycling etching technique essentially consisted of two consecutive processes motivated at Si surfaces, where the cycling reactions were started with an induced etching of Si through copper (Cu) deposition, and followed by the spontaneous elimination of deposited Cu, which acted as a barrier for succeeding dissolution of Si. Anisotropic etching of Si could be sustained as long as these two competitive reactions stayed in dynamic balance, which were accompanied with the gradual variations of slanted angle in formed pyramidal structures. Detailed examinations of morphological evolutions, etching kinetics and formation mechanism were performed, which validated the distinct etching model under the cycling reactions introduced at Cu/Si interfaces. In addition, a further attempt to practically realize the organic/inorganic solar cells was undertaken. This was assembled by incorporating two types of pyramid-type textures with *p*-type conductive polymer featuring the *p*-*n* heterojunction with careful interfacial management. This employment was promised for the development of high-performance nanotextured solar cells.

## Material and Methods

### Fabrication of various Si textures

A single-step procedure of Cu-induced cycling etching was conducted to fabricate pyramid-type textures with controlled profiles. Prior to etching reaction, the monocrystalline Si substrates with (100), (110) or (111) orientation were cleaned through the regular ultrasonication in acetone, isopropyl alcohol (IPA) and deionized water (DI) water. After drying with N_2_ gas, the substrates were dipped into the mixed solutions containing CuSO_4_ (0.01 M), HF (1.2 M) and various concentrations of H_2_O_2_ (0.14 M-0.9 M) for 15 min at 40 °C. Likewise, nanowire-based textures were fabricated using a conventional MaCE reaction under the mixed solutions containing AgNO_3_ (0.02 M) and HF (4.5 M) at 40 °C. After rinsing with DI water several times, the residual Cu nanoparticles were removed completely by dipping the as-prepared samples in a concentrated HNO_3_ (65%) solution for 40 s and then rinsed with DI water several times.

### Device construction

The hybrid solar cells were employed with the *n*-type monocrystalline Si substrates (Resistivity = 1–10 Ωcm). First, the as-prepared textured substrates with sizes of 2 cm by 2 cm were dipped in the dilute hydrofluoric acid (2%) for 2 min in order to completely removing the native oxide grown on Si surfaces. Subsequently, the substrates were rinsed with DI water and then dried with gentle N_2_ gas. 200-nm-thick Al layer was then deposited on the back side of Si substrates with electron-gun evaporator to serve as back electrode. After the construction of Al electrode, Si substrates were transferred to a home-made chamber with controlled humidity of 60% for 2 hr in order to grow an extremely thin layer of Si oxide (1~3 nm) on the surfaces of texturized structures. In addition, a recent literature reported that a very thin layer of Si oxide could also effectively passivate Si surfaces^13^. Next, a PEDOT:PSS dispersion (Clevios PH1000) was spun-coated onto the texturized surfaces at constant speed of 3000 rpm for 1 min and then heated at 120 °C for 15 min under the ambient atmosphere. Finally, a conventional ITO glass (7Ω/square) was gently placed on the Si substrates coated with PEDOT:PSS polymeric layers.

### Characterizations

Morphologies of as-prepared Si textures were characterized using a field emission scanning electron microscopy (SEM, LEO 1530). Etching rates of Si were carefully estimated by measuring the weight loss of Si substrates under various concentrations of H_2_O_2_ reactants after conducting a Cu-induced cycling etching. Cell performances were measured under a standard AM 1.5 G solar simulator equipped with *J*-*V* measurement system (Keithley 2400). Spatial voltage characterizations of fabricated hybrid solar cells with respect to the light illumination (laser diode with wavelength of 450 nm and output power of 5 mW) were performed with LBIV measurement system (LiveStrong Optoelectronics).

## Results and Discussion

**Figure 1:**
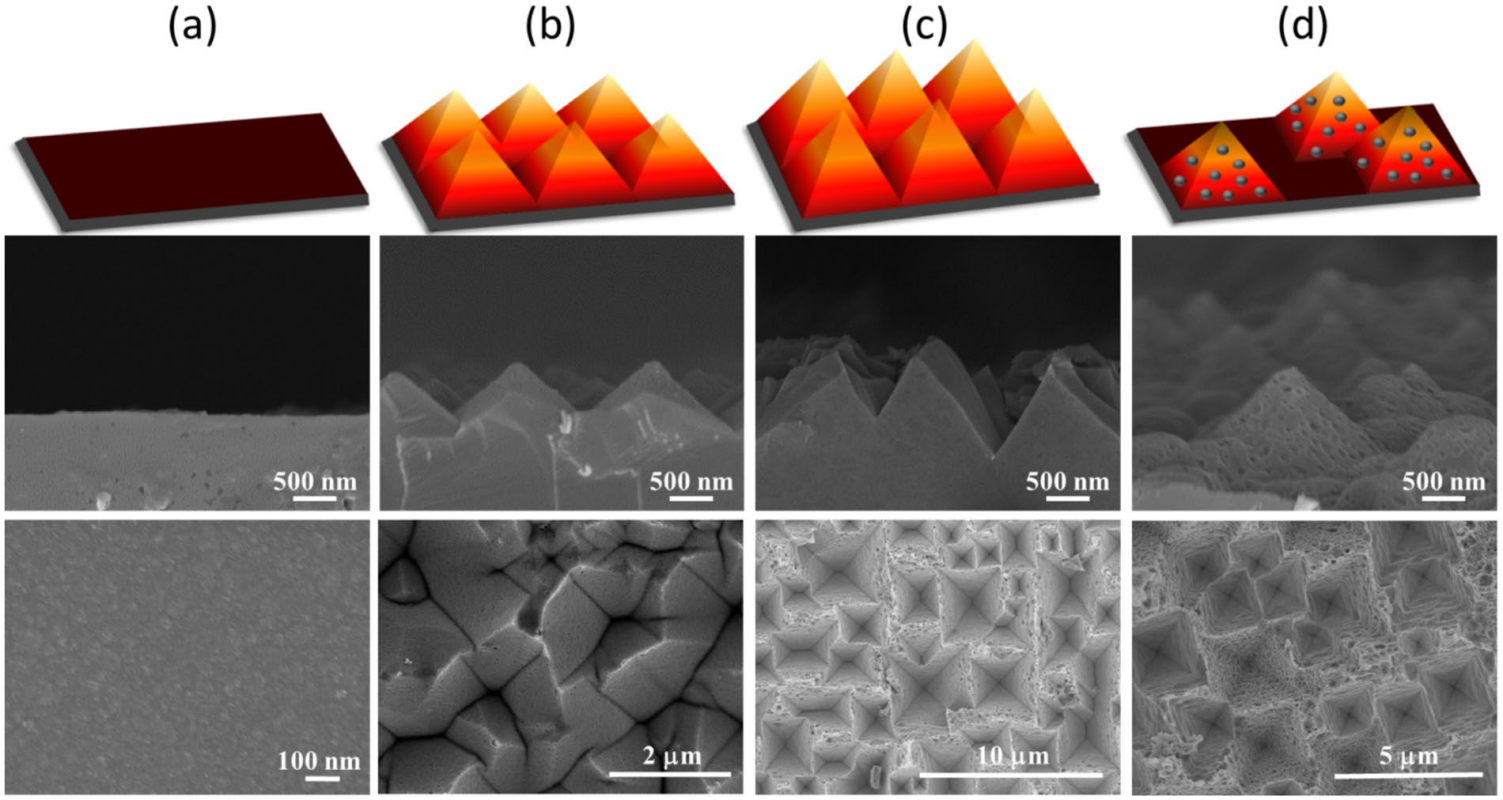
Schematic illustration, cross-sectional view and top view of SEM graphs of Si textures (from top to bottom) obtained from various concentrations of H_2_O_2_ upon Cu-induced cycling etching: (**a**) [H_2_O_2_] = 0.14 M, (**b**) [H_2_O_2_] = 0.53 M, (**c**) [H_2_O_2_] = 0.65 M and (**d**) [H_2_O_2_] = 0.90 M.

Morphological evolutions of Cu-induced cycling etching could be observed in Fig. 1, where the involved concentrations of H_2_O_2_ were gradually increased from 0.14 M, 0.53 M, 0.65 M and 0.90 M, respectively. These results manifested that the strong correlation of etching profiles with H_2_O_2_ concentrations. Specifically, at low H_2_O_2_ concentration (0.14 M), Si textures could be hardly found on the Si surfaces after conducting etching process, as evidenced in both cross-sectional and top view of SEM observations in Fig. 1(a). With the addition of H_2_O_2_ up to 0.53 M, etching profiles were dramatically altered. These features were believed to the overall contributions from cycling reactions induced through the Cu deposition and dissolution. It further clearly implied that the etching characteristics were dramatically transited from the nearly isotropic features toward anisotropic profiles depending on the involvement of H_2_O_2_ concentrations. The formation of pyramid-shaped textures could be clearly observed in Fig. 1(b). With the continued increase of H_2_O_2_ concentration reaching 0.65 M, texturization of Si was accelerated due to the existence of abundant H_2_O_2_ for proceeding the dissolution process of Cu. leading to the pronounced profiles with deeper etching grooves. Instead, the shapes of etching pores were changed to be inverted-pyramid structures, as particularly imaged in Fig. 1(c). In the case of high concentrated H_2_O_2_ [0.90 M], the uneven and rough surfaces textured by Cu-induced cycling etching could be found, as shown in Fig. 1(d).

**Figure 2:**
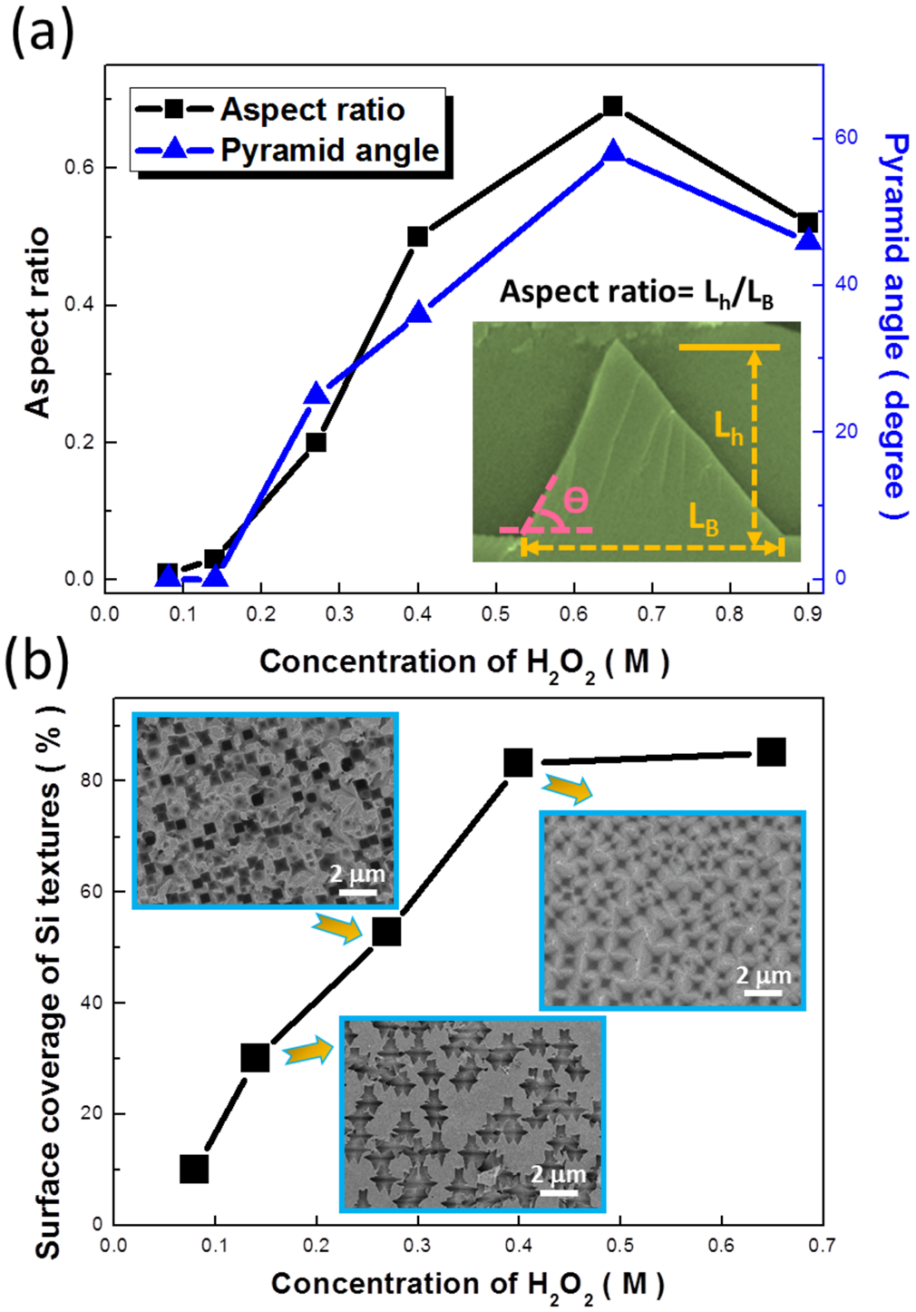
(**a**) Examinations of formed Si pyramid-based textures on both aspect ratios and pyramidal angles with respect to the involved concentrations of H_2_O_2_. (**b**) Dependence of coverage percentage of Si textures on the H_2_O_2_ concentrations. Surface morphologies of corresponded textures were demonstrated in the insert figures.

To gain more insights on the evolution of etching profiles when tuning the concentrations of H_2_O_2_, the aspect ratio and angle of pyramid structures were estimated, as shown in Fig. 2(a), where the pyramid angle (θ) was defined by the angle between the taped sidewalls and base of etched pyramids. Structural estimations were carefully employed from 60 sets of pyramid textures, respectively, and the average values were recorded. Evaluations of structural profiles revealed that the most pronounced aspect ratio of etched textures reached around 0.7 while the H_2_O_2_ concentration of 0.65 M was involved. Since the etching durations of all the tested experiments kept irreverent, it suggested that the formation of such inverted-pyramid features were the kinetically preferable under the involvement of Cu-induced cycling etching. Also, the pyramid angle of texturized structures presented the largest value (58^0^) at the similar concentration of H_2_O_2_. Under such condition, the taped facets of Si textures belonged to <111> orientations according to the lattice configuration of Si crystals. Moreover, one could further observe that the change of aspect ratio with respect to the various concentrations of H_2_O_2_ approximately matched the modulation of pyramid angles, indicating the fact that these two geometrical factors were mutually dependent to the involved H_2_O_2_ contents, as evidenced in Fig. 2(a).

In addition, surface coverage of textures under different H_2_O_2_ concentrations was monitored to examine the fabrication uniformity of utilized etching technique. This was achieved by quantitatively evaluating the relative surface area possessed via the imaging analysis (ImageJ), as presented in Fig. 2(b). The monolithic increase of surface coverage from <10% toward approximately 85% could be found when increasing the concentration of H_2_O_2_ from 0.08 M to 0.45 M, respectively, and then stably saturated at 82–85% upon the H_2_O_2_-invloved circumstance in the range of 0.45 M–0.68 M. It clearly emphasized the influences of H_2_O_2_ content on the extent of texturization uniformity, and further revealed the fabrication window of this etching technique (0.45 M~0.68 M in H_2_O_2_ concentration). Besides, uniformly distributed deposition of Cu nanoclusters covering throughout the Si surfaces upon etching reaction was evidenced, which also acted a significant role for the resulting uniform textures, as evidenced in the Supplementary Information. Combined all these corresponded results from geometrical investigations in Fig. 2(a) and (b), it could be speculated that the controlled formation of pyramidal textures was indeed dominated by the involved reactants with respect to their relative amounts, where the etching features could be intentionally tuned by taking considerations of structural parameters including aspect ratio, taped angle and surface coverage. Furthermore, there must exist the relative amount of H_2_O_2_-HF-CuSO_4_ electrolytes that would response to the reliability and stability of this etching technique.

**Figure 3:**
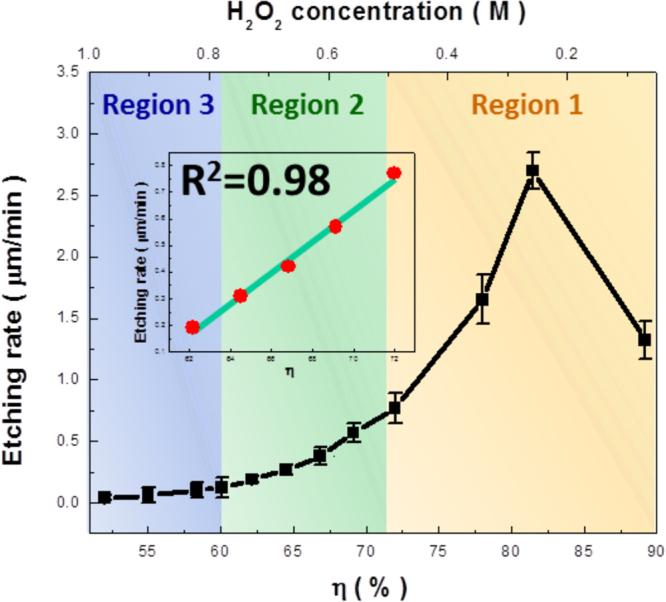
Plot of etching rate with respect to the involved concentration ratio (η) of H_2_O_2_-HF-CuSO_4_ electrolyte system, where the evolution of etching kinetics could be broken into contributions from three separated regions. The insert figure presented the linear regression of etching rates versus the corresponding values of η.

To unveil the influences of reactive compositions on the etching kinetics, the concentration ratio of H_2_O_2_-HF-CuSO_4_ electrolyte system, η, was defined^14,15^,

1 $${\rm{\eta }}=[{\rm{HF}}]/([{{\rm{H}}}_{2}{{\rm{O}}}_{2}]+[{\rm{HF}}]+[{{\rm{CuSO}}}_{4}])$$

where the etching rates of Si substrates depending on the wide spam of η were investigated under a Cu-induced cycling reaction, as presented in Fig. 3. Essentially, evolution of etching kinetics could be broken into contributions from three seperated regions. At Region 1, reaction of Si dissolution was dominantly contributed by the direct displacement of Cu^2+^ ions and Si and followed by dissolution of Si oxide with HF etchants, as expressed by the following equations^16–18^,

2 $${{\rm{Cu}}}^{2+}+2{{\rm{e}}}^{-}\to {\rm{Cu}}$$

3 $${\rm{Si}}+2{{\rm{H}}}_{2}{\rm{O}}\to {{\rm{SiO}}}_{2}+4{{\rm{H}}}^{+}+4{{\rm{e}}}^{-}$$

4 $${{\rm{SiO}}}_{2}+{\rm{HF}}\to {{\rm{H}}}_{2}{{\rm{SiF}}}_{6}+2{{\rm{H}}}_{2}{\rm{O}}$$

In such condition, Cu nanocluters were favorably grown on Si surfaces through a galvanic displacement between Cu^2+^ ions and Si due to the fact that the electronegativity of Cu (1.9) is higher than that of Si (1.8)^16^. Nucleation of Cu nanoclusters were taken place by withdrawing the electrons from Si substrates and accompanied with the direct oxidation of Si surfaces in contact with Cu^2+^ ions. The involving oxidation of Si followed by oxide dissolution with HF etchants was essentially isotropic with no preferred orientation, leaving a Si surfaces with fine pores after removing the deposited Cu, as presented in Fig. 1(a). Also, the nature of electroless deposition was highly subjected to the diffusion-limited circumstances. Namely, it could be speculated that the deposition reaction tended to slow down while the dense Cu layers were generated. Metal deposition was eventually terminated because the oxide-dissolution species, i.e., F^−^ ions were incapable of reaching the Cu/Si interfaces for the initiation of removing the grown oxide^19^. Meanwhile, with the gradual reduction of η (concentration of H_2_O_2_ over 0.30 M), the introduction of competitive dissolution reaction of Cu in addition to the active growth of Cu was taken place, which could be given by,

5 $${\rm{Cu}}+{{\rm{H}}}_{2}{{\rm{O}}}_{2}+2{{\rm{H}}}^{+}\to 2{{\rm{H}}}_{2}{\rm{O}}+{{\rm{Cu}}}^{2+}$$

Competition of Cu dissolution against the reduction of Cu^2+^ ions was involved, responding to the substantial decrease of dissolution rates of Si, as shown in the Region 1 (η < 80%) of Fig. 3. It should be noted that the overall reaction still favored the continued deposition of Cu since in this moment the involved H_2_O_2_ regents remained insufficient for the complete removal of grown Cu clusters.

By decreasing η toward 72% (the addition of H_2_O_2_ over 0.50 M), the competitive processes of Cu deposition and dissolution reached a quasi-stable configuration; that is, the kinetic balance of these two reactions was established. This resulted in the monolithic decrease of etching rates with respect to the reduction of η, where the etching evolution was moved to Region 2 in Fig. 3. Interestingly, the etching characteristic in Region 2 explicitly corresponded to the linear correlation with composition ratio, η, which was supported with the sound regression value of R^2^ = 0.98. It implied that the etching rates in Region 2 could be well controlled by tuning the molar ratio of reactants in terms of η. In addition, distinct morphologies of Si textures in either pyramidal shape [Fig. 1(b)] or inverted-pyramid features [Fig. 1(c)] appeared to be the dominant structures with η of 69% and 64%, respectively. In Region 3, the involvement of substantial concentrations of H_2_O_2_ hindered the continued deposition of Cu on Si and thereby, etching of Si was almost ceased with the variation of η. These features led to the unchanged etching rate responding to the decrease of η, where only a few irregularly distributed Si textures with porous sidewalls were created based on the morphological observation shown in Fig. 1(d).

**Figure 4:**
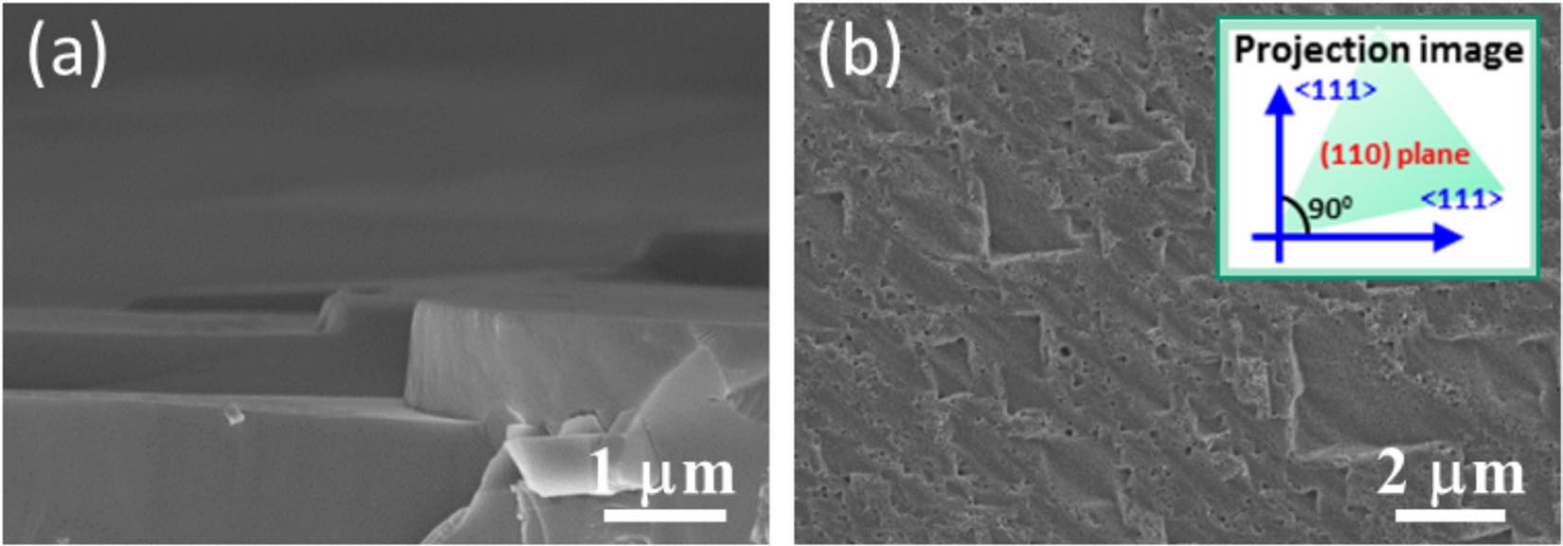
(**a**) Cross-sectional (**b**) top-view SEM images of (110)-oriented Si substrates texturized with Cu-induced cycling reaction. The inset figure shown in Fig. 4(b) visualized the two principle axes of etched facets projected on the (110) plane, showing the orthogonal configurations along <111> orientations.

To explore the etching orientations obtained from Cu-induced cycling reaction, various oriented Si substrates including (110) and (111) single-crystalline wafers were employed. The cross-sectional SEM investigations of textured surfaces created from (110)-oriented substrates were presented in Fig. 4(a), demonstrating the etched trenches vertically to the substrate plane with well regularity. Interestingly, the etching depth was approximately consistent with the results prepared with (100)-oriented wafers [Fig. 1(b)] after experiencing the similar etching durations; instead the etched profiles were dramatically different. In addition, no obvious etched structures could be found on the surfaces of Si (111) substrates, as shown in the Supplementary Information. It implied the appearance of preferential etching direction via such Cu-induced cycling reaction regardless of the crystalline orientation of utilized Si substrates. To understand it further, the crystallographic projection of formed Si textures from Si (110) substrates was examined^20^, as presented in Fig. 4(b). This was performed by characterizing the majority of textures’ facets from the top-view SEM observations. Projection of two major directions onto (110) plane followed two principle axes which were perpendicular to each other, as evidenced in the insert figure of Fig. 4(b). It could be concluded that these directions belonged to <111> orientations and agreed well with the result of profile examinations shown in Figs 1 and 2.

**Figure 5:**
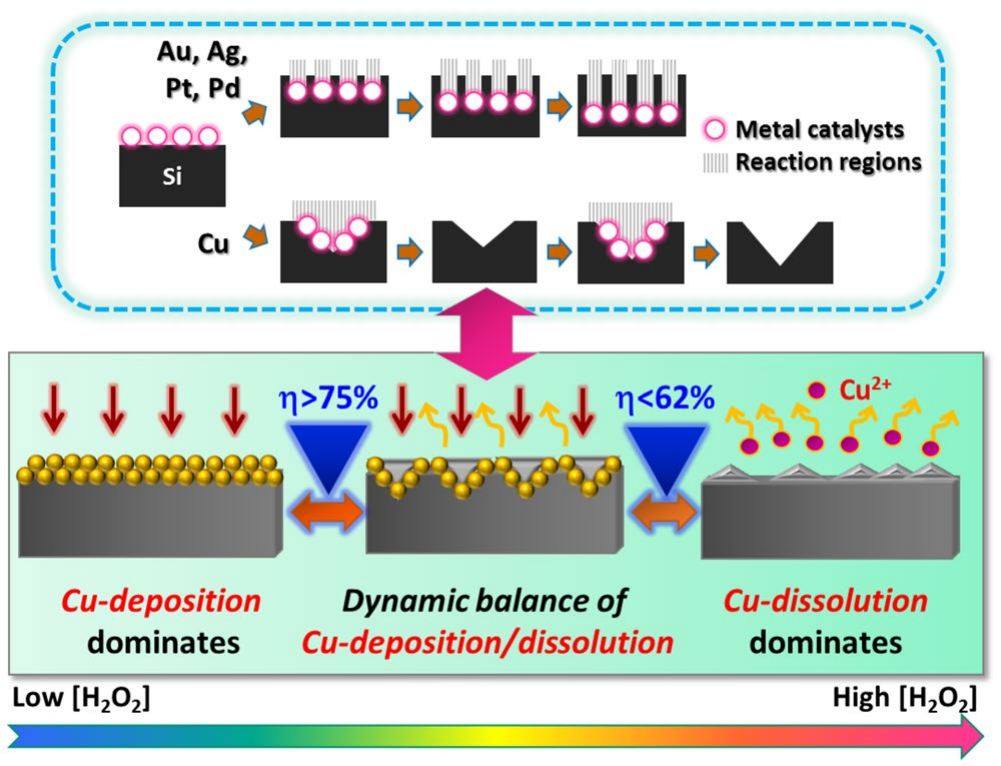
Schematic models presented the dominant reactions contributing to the morphological evolution of Si surfaces under various ranges of composition ratio (η) of reactive species. The fundamental difference of etching path involved with metal catalysts was also schematically illustrated to clarify the role of Cu upon initiating a distinct metal-induced cycling reaction on texturizing Si.

We attempted to elucidate the formation mechanism of pyramid-type Si textures uniformly covered on the Si surfaces, as illustrated in Fig. 5. It has been extensively reported that the catalytic etching of Si assisted by the defined metal structures could result in the formation of one-dimensional Si nanostructures^21^. Etching conditions, such as involved reactive species, reaction temperature and time might be varied depending on the desired lengths or dimensions of etched products^22,23^. Still, the formed nanostructures were consistently one-dimensional in geometry wherever different catalytic materials, such as Au, Ag, Pt and Pd, were employed, respectively. In fact, the utilized catalysts, as aforementioned, could stand for a lengthy etching process. In such case, the decomposition of H_2_O_2_ oxidants was taken place closely at catalyst surfaces, where the generated holes preferentially constituted the dissolution of Si beneath catalytic sites through the hole-injection process in HF-involved environment^24^. As long as these catalysts paved the primary route for Si etching, directional dissolution of Si with one-dimensional geometry was energetically predominant since any change of etching direction inevitably consumed the additional energy in some extent.

Nevertheless, the utilization of Cu as catalysts was believed to alternatively pave the unique way for the texturization of Si. While the molar ratios of H_2_O_2_-HF-CuSO_4_ were within the Region 2 [Fig. 3], the as-deposited Cu nanoclusters would be rapidly dissolved in the H_2_O_2_ involved solutions prior to continued growth of Cu layers, as depicted in Fig. 5. This step was quite crucial for the intended modulation of Si surfaces because it boosted the newly arrived Cu^2+^ ions to reside and nucleate at texturized Si surfaces, which had experienced the electroless deposition/dissolution of Cu. According to the pioneer study on the electrochemical fabrication of porous Si reported by V. Lehmann^25^, the accumulated holes introduced by an external electric field preferentially injected into the existed pits which possessed the small curvature radius upon electrochemical etching. Thus, it could be understood that the hole injections supplied from Cu^2+^ ions were facilitated in the vicinity of textured sites rather than the un-etched regions, and the effective galvanic deposition of Cu could be expected due to the fact that the exchange of carriers were locally restrained at texturized Si surfaces. Such redeposition of Cu was accompanied with breaking the surface bonding of Si that responded to dissolution process. Through the cycling reactions induced by the Cu deposition and dissolution, evolution of etching characteristics was dramatically transited from nearly isotropic features toward anisotropic profiles. Accordingly, the formation of intermediate pyramidal textures could be clearly observed in Fig. 1(b), where the corresponding n was 69% (C_H2O2_ = 0.53 M).

On the other hand, with the reduction of η at 64% (C_H2O2_ = 0.65 M) in process, the inverted-pyramid Si textures with pronounced aspect ratio was formed owing to the existence of abundant H_2_O_2_ for accelerating the cycling reaction of Cu deposition/dissolution. It should be noted that the etching rate of the Cu-induced cycling etching was more likely to be determined by breaking the surficial bonding of Si through an electroless displacement of Si with Cu^26^. Since there were no additional bias such as electric field or heat applied on this system, etching of Si was preferentially taken place at the energy-favorable sites in order to minimizing the energy barrier that was responsible for nucleation. Considering the crystalline configurations of Si, it has been reported that <111> orientation constitutes the stable bonding configuration in Si crystals according to the model of surficial bonds^27^. This explained the successive formation inverted-pyramid shapes of textures whose {111} planes left on the texture sidewalls essentially behaved as the etching-stop lattice configuration. Based on the origin of texture preparation, it should be emphasized that the fabrication uniformity of such etching technique highly relied on the fluid mechanics of aqueous systems as the overall processes involved the multiplex reactions localized in the vicinity of Cu nanoclusters^28^. Actually, it was found that no obvious textures could be created under the etching condition without applying a magnetic stir, as shown in the Supplementary Information. Moreover, with an introduction of agitation motion for aqueous regents driven by the optimal stirring condition (stir rate = 250 rpm), it could readily facilitate the cycling reactions of Cu that was responsible for the anisotropic texturization of Si, and thereby responded to the uniform textures covered throughout the Si surfaces.

**Figure 6:**
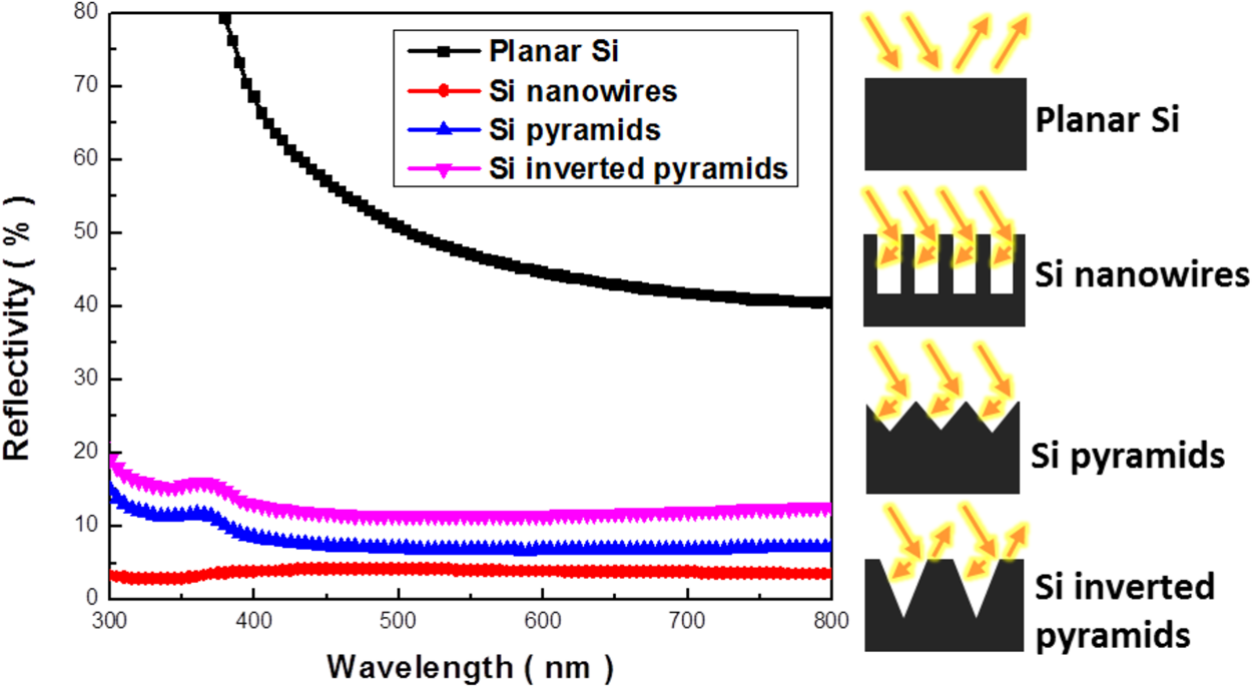
Spectral reflectance of Si textures with various profiles: planar Si (untexturized Si), Si nanowire arrays made with conventional Ag-based MaCE process, pyramidal and inverted-pyramidal arrays prepared with Cu-induced cycling etching, respectively.

In addition to the structural examinations along with mechanism study, the spectral reflectance of Si textures with various shapes were investigated, as demonstrated in Fig. 6. Here, nanowire samples and two types of pyramidal structures were prepared with conventional Ag-based MaCE and Cu-induced cycling etching, respectively. Compared with planar Si whose reflectivity was spectrally higher than 40% within the broadband illuminations from 300 nm to 800 nm, all other texturized architectures demonstrated the greatly suppressed refractivity with average value of 3.7% from Si nanowires, 8.9% from Si pyramids and 13.6% from Si inverted pyramids, respectively. It has been reported that the illuminated lights encountered the multiple scattering conditions with the nanoscale features whose dimensions were less than the wavelengths of incoming light^29^. This qualitatively explained the most pronounced suppression of light reflection from the case of nanowire-based textures. Aside from the characteristics of light scattering, the effective refractive index *n(z)* of textures can be described according to effective medium theory, as expressed below^30^,

6 $${\rm{n}}({\rm{z}})={\{{\rm{f}}({\rm{z}}){{{\rm{n}}}_{{\rm{Si}}}}^{{\rm{q}}}+[1-{\rm{f}}({\rm{z}})]{{{\rm{n}}}_{{\rm{air}}}}^{{\rm{q}}}\}}^{1/{\rm{q}}},{\rm{q}}=2/3$$

in which z represented the texture thickness, *f(z)* the fraction of Si, *n*_*Si*_ and *n*_*air*_ the refractive indices of Si and air, respectively. Accordingly, the subwavelength pyramidal textures possessed the intermediate value of effective refractive index right between Si substrates and surrounding air, which introduced the gradual change of refractive index responding to the pyramidal geometry of Si textures that minimized the possible optical loss at Si/air interfaces, thus leading to the suppressed reflectivity within the broadband solar regions. On the other hand, the distinct optical response associated with spectral reflectivity of light appeared in Si pyramids (average reflectivity = 8.9%) and inverted pyramids (average reflectivity = 13.6%), which could be further elucidated through the schematic illustration of optical path shown in Fig. 6. Accordingly, the additional interfacial reflection of light could be actually motivated at the top surfaces of inverted pyramidal textures, eventually limiting their antireflection capability for trapping the incoming light.

**Figure 7:**
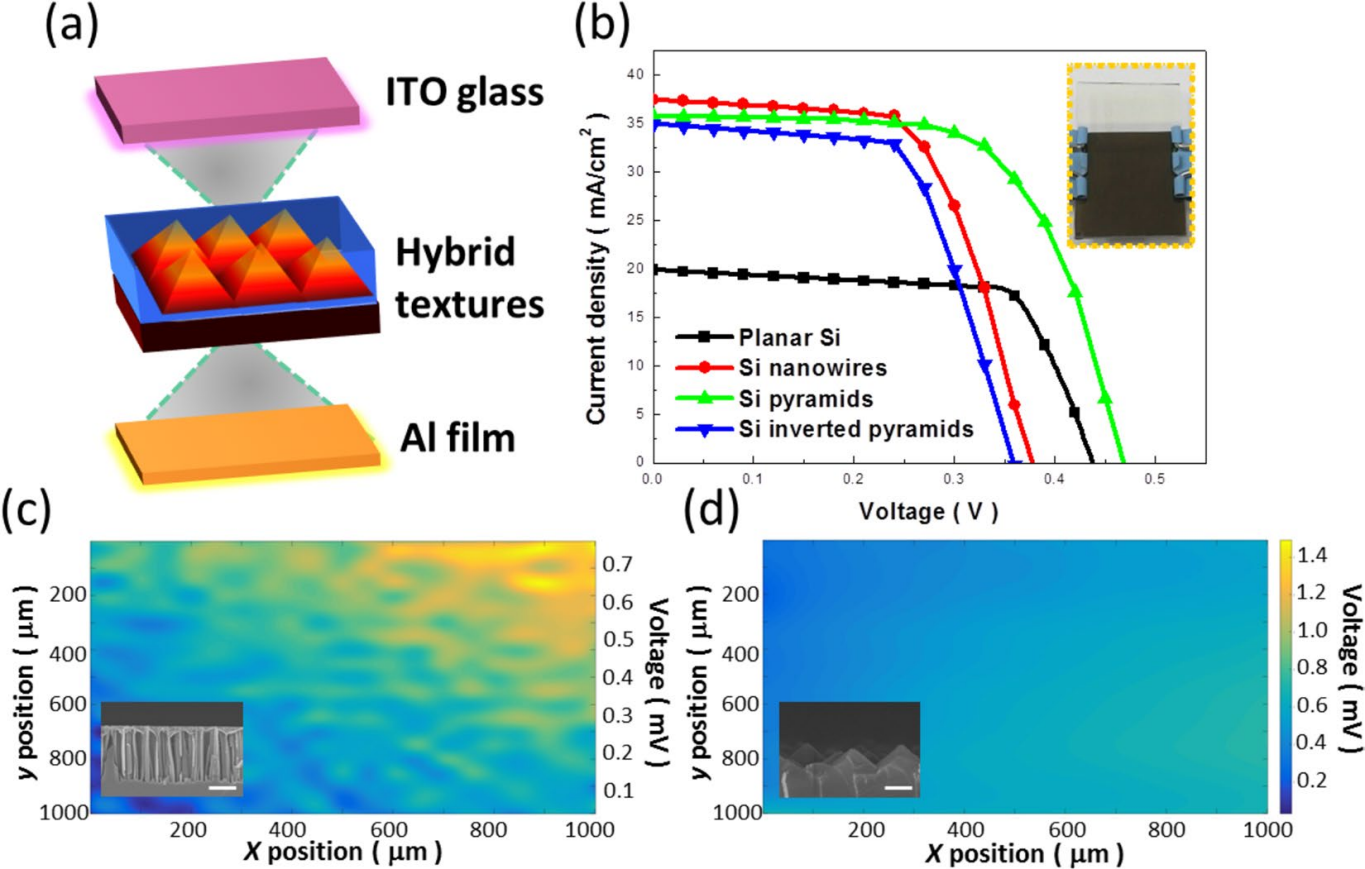
(**a**) Schematic drawing of device structure for the construction of hybrid solar cells. (**b**) Measured *J*-*V* photovoltaic results of hybrid solar cells with a variety of tailored Si textures. (**c**) LBIV characterization of voltage fluctuations in response to the illuminated mapping of laser beam: (**c**) Nanowire-based and (**d**) Pyramid-based hybrid solar cells. The inserted SEM images indicated the practical deposition of p-type polymeric layer on the Si textures, respectively.

To explore PV effects of two types of pyramidal Si textures, the *p*-*n* heterostructures were created by incorporating the *n*-type Si textures in conjunction with *p*-type conductive polymers, as depicted in Fig. 7(a). Conventional ITO glass and a deposited Al layer as top and bottom electrode were introduced to build up the basic electric connection, where the whole procedures of cell construction along with the characterizations of PV performance were described in the Experimental Section. Figure 7(b) presented the measured current (*J*)-voltage (*V*) curves of sandwich-type hybrid solar cells. Impacts of surface texturization on the cell performance could be clearly revealed through the modulations of texturized structures, including bare substrates (planar Si) as references, nanowires, pyramidal and inverted-pyramid textures, respectively, while holding all other fabrication procedures with consistency. The major PV parameters extracted from *J*-*V* measurements were demonstrated in Table 1. In addition, the measured photovoltaic result of hybrid solar cells texturized with conventional alkaline-based etching could be found in the Supplementary Information. Among them, planar Si based solar cells correlated with the lowest cell efficiency (6%), owing to the limited value of short-circuit current density (*J*_*sc*_). These results could be interpreted by the high reflectivity of planar Si that significantly hindered the effective absorption of photons for supplying photogenerated carriers^31,32^.

**Table 1:** Average reflectivity (R_avg_), open circuit voltage (V_OC_), short circuit current density (J_SC_), fill factor (FF) and conversion efficiency of four different hybrid solar cells of Si nanocrystals.

Texturized structure	R_avg._ [%]	V_OC_ [V]	J_SC_ [mA/cm^2^]	FF [%]	Efficiency [%]
Planar Si	53.2	0.44	19.90	68.95	6.0
Si nanowires	3.7	0.38	37.51	62.04	8.8
Si pyramids	8.9	0.47	35.73	64.54	10.7
Si inverted pyramids	13.6	0.36	34.92	62.97	7.9

With the experience of texturization procedures, the values of *J*_*sc*_ in three texturized solar cells were evidently improved. The highest *J*_*sc*_ appeared in nanowire-based hybrid solar cells, which corresponded well to their lowest light reflectivity presented in Fig. 6. Nevertheless, the nature of high aspect ratio from nanowire architectures inevitably impeded the uniform coverage of p-type polymer with underlying nanowire-involved textures, which reflected to the reduced value of open-circuit voltage (*V*_*OC*_) due to the poor deposition coverage of polymeric layers^33^. Meanwhile, the result of finite junction area of polymer/nanowire heterostructures was encountered, and responded to the low fill factor (*FF*) resulting from the insufficient charge transport pathways of photoexcited carriers^33,34^. These combined effects compromised against the great improvement of *J*_*sc*_ and eventually led to the cell efficiency of 8.8%. Such severe trade-off condition between pursuing low optical reflectivity and sustaining large-area heterojunction with sound uniformity could be efficiently relieved by introducing the pyramidal textures on cell construction. These textures not only maintained the sound antireflection characteristics, but further eased the fabrication burden in terms of the creation of uniform *p*-*n* heterojunctions, which therefore possessed the leading conversion efficiency of 10.7%, approximately 1.8 times and beyond 1.2 times greater than that of untexturized (planar Si) and nanowire-based solar cells, respectively. In addition, over 20 sets of devices were fabricated and measured, showing the reliable conversion efficiency of fabricated solar cells.

Moreover, the origin of performance improvement based on pyramid-involved hybrid cells could be further supported by the spatial characterizations with light beam induced voltage (LBIV) analysis^35^, as compared in Fig. 7(c) and (d). By mapping the voltage fluctuations responded to the scanning of light beam, one could apparently observe the abrupt change of instant voltage with respect to the surficial positions of nanowire-based hybrid solar cells [Fig. 7(c)], which strongly correlated with the structural non-uniformities or defective sites occurred in the cell structures. Cross-sectional SEM image of polymer/nanowire junction was displayed in the insert figure of Fig. 7(c), evidencing the fact that the polymeric layer was predominantly deposited on the top of Si nanowires rather than covering on the nanowires’ sidewalls. It should be noted that the hybrid solar cells with the incorporation of inverted pyramids as textures suffered the similar problems against the resulting efficiency as they demonstrated both the comparable *V*_*oc*_ and *FF*, as found in Table 1. By contrast, the comparably uniform and invariant LBIV image under a large-area scanning of light beam was visualized in pyramid-based hybrid solar cells [Fig. 7(d)], again validated the improvement of creating *p*-*n* heterostructures as well as the resulting cell efficiency.

## Conclusions

In conclusion, we have explored the role of Cu catalysts on the modulations of Si textures in the presence of H_2_O_2_-HF-CuSO_4_ aqueous system, where the formation of both pyramidal and inverted-pyramid profiles with controlled aspect ratio and coverage uniformity could be tailored. This etching technique, introduced by the cycling reactions of Cu deposition and dissolution, promised a compelling capability for texturizing Si surfaces rather than the generic unidirectional nanowire-based structures, which were validated as promising design for advanced photovoltaic applications. Our results explicitly revealed the improved conversion efficiency of pyramid-based hybrid solar cells by means of reducing the defective sites essentially arisen from the contact non-uniformities between organic *p*-type layers and inorganic *n*-type nanostructures. This approach enabled to provide a unique opportunity for the understanding of nanoscale etching which remained unclear from the existing techniques and related studies, and might further extend the potential applications for various functional devices.

## Electronic supplementary material

Supplementary Information
