# Supplementary material for: Hybrid black silicon solar cells textured with the interplay of copper-induced galvanic displacement
Source: Sci Rep. 2017 Dec 7;7:17177. doi: 10.1038/s41598-017-17516-6 (PMC5719426; doi:10.1038/s41598-017-17516-6)
Supplement: Supplementary file 1 — Supplementary Information [file 41598_2017_17516_MOESM1_ESM.pdf]

Supplementary Information

Hybrid black silicon solar cells textured with the interplay of copper-induced galvanic displacement

Jheng-Yi Li, Chia-Hsiang Hung, and Chia-Yun Chen

Department of Materials Science and Engineering, National Cheng Kung University, Tainan 701, Taiwan

S1 Morphologies of Cu nanoclusters formed through the galvanic displacement

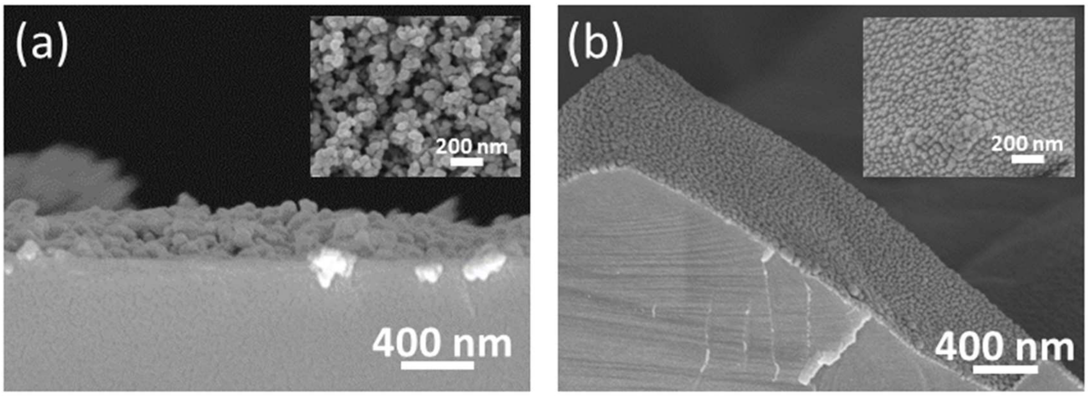

Figure S1 Representative cross-sectional SEM images of etched textures prepared with Cu-induced cycling etching without removing the grown Cu nanoclusters: (a) [H_2O_2]= 0.14 M and (b) [H_2O_2]= 0.53 M. The insert figures present the top-view SEM image of corresponded textures. The results indicate the distinct morphologies of Cu deposition depending on the involved [H_2O_2] concentrations. With [H_2O_2] of 0.14 M, dense Cu nanoclusters behave as a continuous layer with thickness of 200-400 nm covering throughout on the Si surfaces, leaving no obvious Si textures left. On the other hand, with [H_2O_2] of 0.53 M, the distributed Cu nanoclusters with remarkable uniformity covering on the Si textures are formed. These features are evidenced to act as significant role for facilitating the cycling reactions of Cu deposition and dissolution, and thereby initiate the formation of desired pyramidal textures.

S2 Etching behaviors on single-crystalline Si (111) substrates

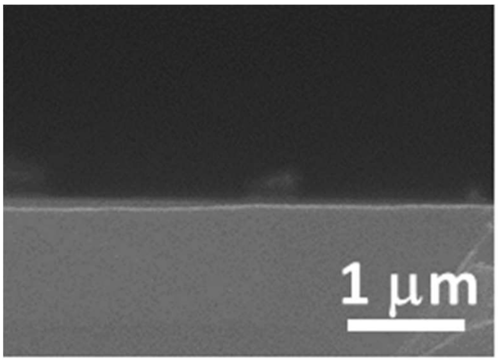

Figure S2 (a) Representative cross-sectional SEM image of Si (111) surfaces while undergoing the Cu-induced cycling etching. This finding indicates that the involved etching technique is highly dependent on the crystallographic orientation of applied Si substrates. Also, it further supports the clarification of orientated etching presented in the main text, where the {111} planes hold the etching stop configuration.

S3 Investigations of stirring contributions to the fabrication uniformity of Cu-induced cycling etching

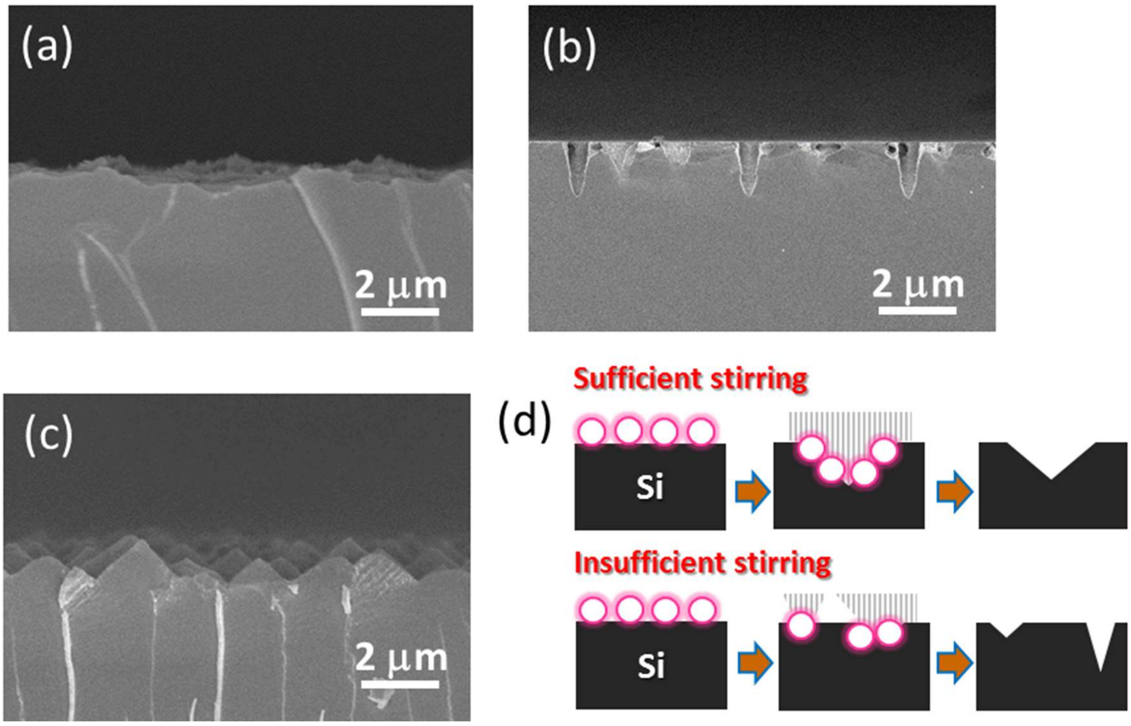

Figure S3 (a) Investigations of stirring rates during etching process on the formation uniformity of Si pyramidal textures: Cross-sectional SEM images of Si textures with stirring rate of (a) 0 rpm, (b) 120 rpm and (c) 250 rpm. Compared with rather distributed and irregular textures made with insufficient stirring rates [Figure S3(a)

and 3(b)], the uniform formation of pyramidal textures were achieved [Figure S3(c)] due to the involvement of sufficient agitation motion in aqueous system that facilitates the Cu-induced cycling reactions, where the underlying etching transitions can be interpreted by the comparative illustrations shown in Figure S3(d).

S4 Light reflectivity and J-V measured result of hybrid solar cells texturized with conventional alkaline-based etching

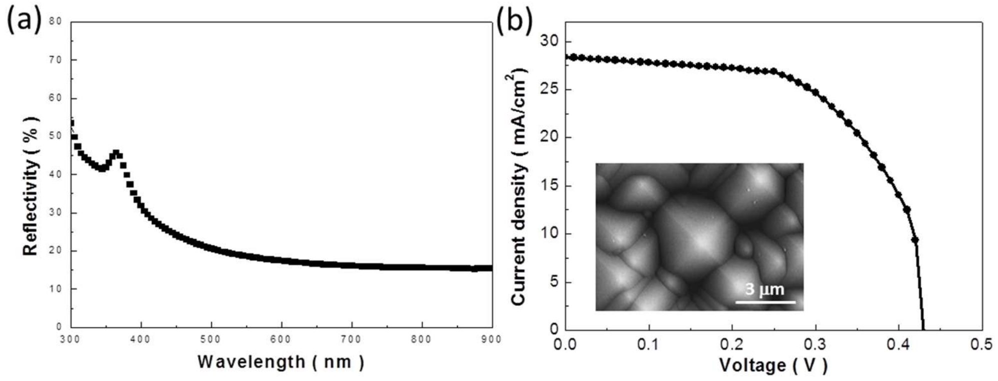

Figure S4 (a) Spectral reflectance and (b) measured J-V photovoltaic results of hybrid solar cells texturized with conventional alkaline-based etching. The average reflectivity of such texturized structures is 18.9%, and the resulting conversion efficiency of hybrid solar cells is 7.3%. The lower conversion efficiency in comparison with pyramid-based textures made with Cu-induced cycling etching can be attributed to the comparably higher light reflectivity.
